# Supplementary material for: Genotype-by-inhibitor interactions to dissect enterovirus replication
Source: Nat Commun. 2026 Apr 11;17:5089. doi: 10.1038/s41467-026-71900-3 (PMC13247092; doi:10.1038/s41467-026-71900-3)
Supplement: Supplementary file 1 — Supplementary Information [file 41467_2026_71900_MOESM1_ESM.pdf]

Supplementary Information for "Genotype-by-Inhibitor Interactions to Dissect Enterovirus Replication", William Bakhache, Walker Symonds-Orr, and Patrick T. Dolan

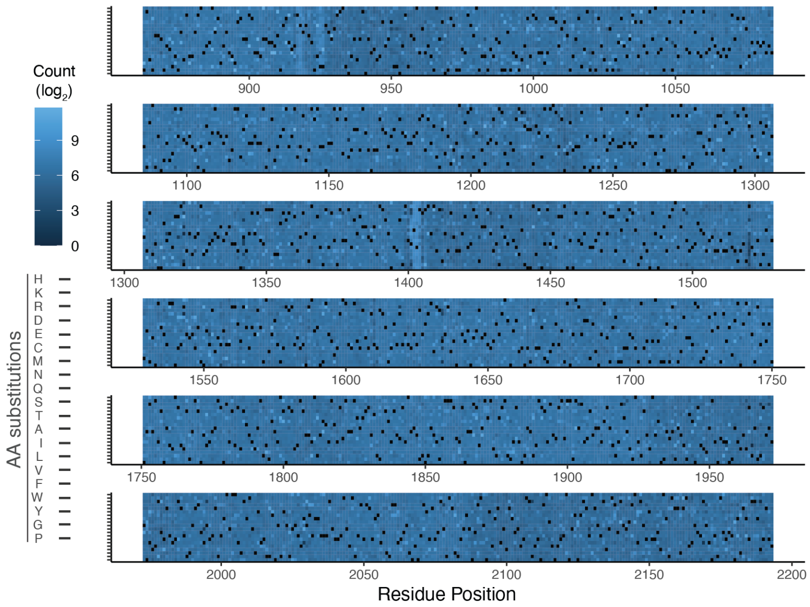

Supplementary Figure 1: Plasmid Deep Mutational Scanning Library Balance. *Heatmap showing log_2-transformed mutation counts (pseudocount +1) across the EV-A71 replication proteins in the plasmid input library. Black squares represent the wild-type sequence.*

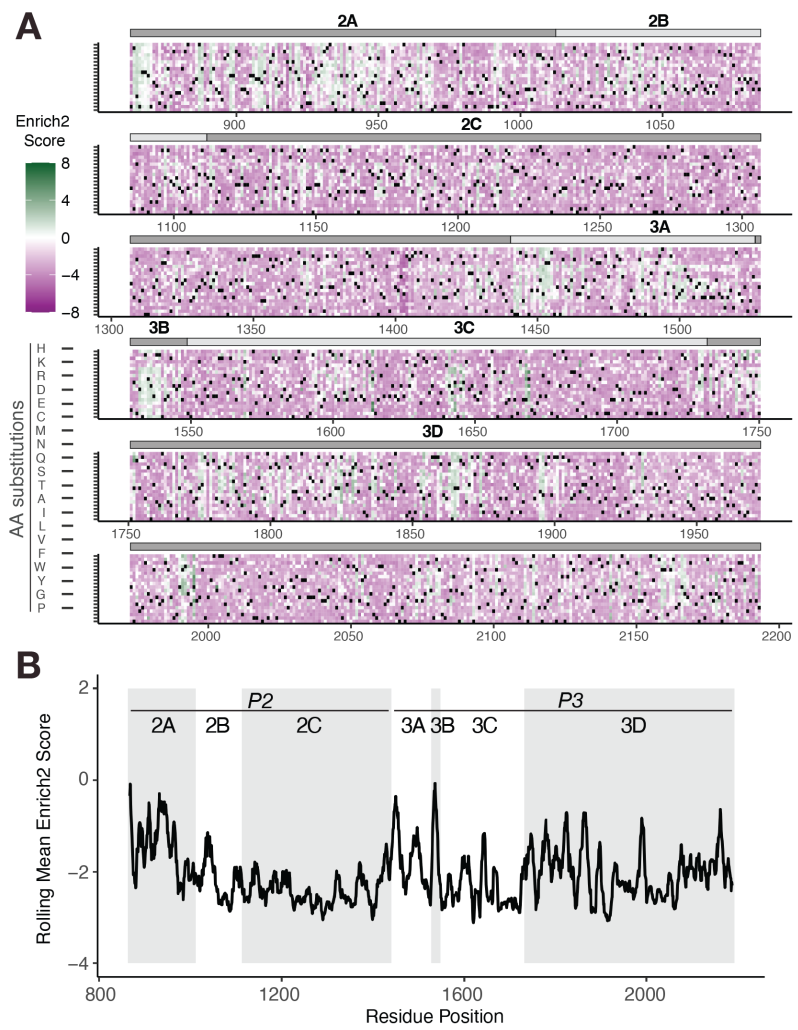

Supplementary Figure 2: Deep Mutational Scanning of EV-A71 Replication Proteins *(A) Heatmap showing the Enrich2 score of mutations across the EV-A71 replication proteins. Black squares represent the wildtype sequence. (B) Line plot showing the mean Enrich2 score using a 10 amino acid sliding window across the EV-A71 replication proteins.*

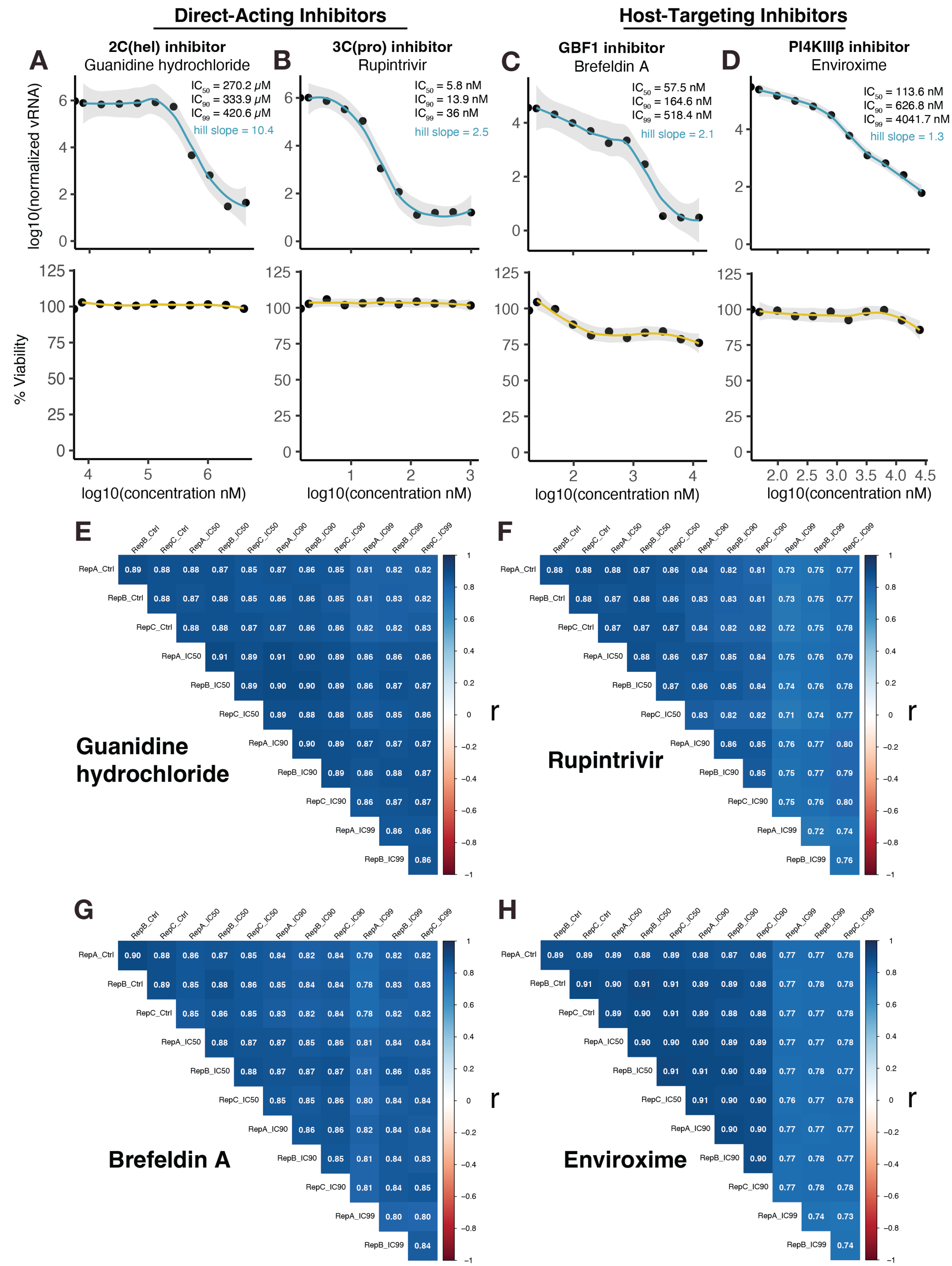
Direct-Acting Inhibitors
Host-Targeting Inhibitors
A
2C(hel) inhibitor
Guanidine hydrochloride
IC50 = 270.2 μM
IC90 = 333.9 μM
IC99 = 420.6 μM
hill slope = 10.4
B
3C(pro) inhibitor
Rupintrivir
IC50 = 5.8 nM
IC90 = 13.9 nM
IC99 = 36 nM
hill slope = 2.5
C
GBF1 inhibitor
Brefeldin A
IC50 = 57.5 nM
IC90 = 164.6 nM
IC99 = 518.4 nM
hill slope = 2.1
D
PI4KIIIβ inhibitor
Enviroxime
IC50 = 113.6 nM
IC90 = 626.8 nM
IC99 = 4041.7 nM
hill slope = 1.3
log10(normalized vRNA)
% Viability
log10(concentration nM)
E
Guanidine hydrochloride
F
Rupintrivir
G
Brefeldin A
H
Enviroxime
r

Supplementary Figure 3: Effect of Inhibitors on EV-A71 Replication and Cell Viability, and Reproducibility of Enrich2 Scores *Dose-response curves showing the effect of* ***(A)*** *Guanidine hydrochloride,* ***(B)*** *Rupintrivir,* ***(C)*** *Brefeldin A, and* ***(D)*** *Enviroxime on EV-A71 RNA levels (top panels; mean of three biological replicates) and cell viability (bottom panels; mean of two biological replicates). Pearson correlation matrices showing reproducibility of Enrich2 scores across biological replicates and under different inhibitory concentrations for (E) Guanidine hydrochloride, (F) Rupintrivir, (G) Brefeldin A, and (H) Enviroxime.*

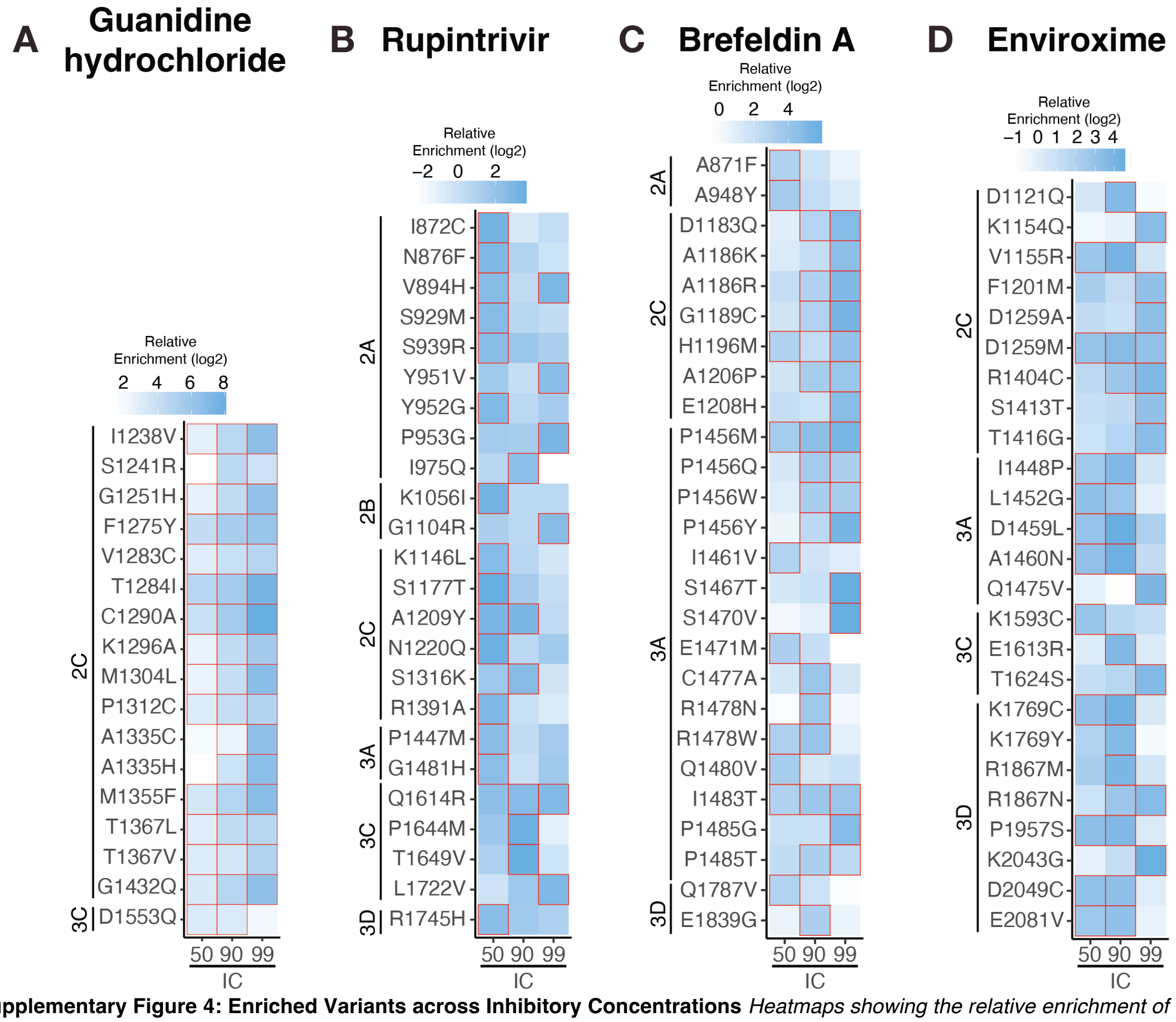

Supplementary Figure 4: Enriched Variants across Inhibitory Concentrations *Heatmaps showing the relative enrichment of the top variants identified at IC_{50}, IC_{90}, and IC_{99} for* ***(A)*** *Guanidine hydrochloride,* ***(B)*** *Rupintrivir,* ***(C)*** *Brefeldin A, and* ***(D)*** *Enviroxime. For each inhibitor, the top 10 enriched variants per inhibitory concentration were included, with overlapping variants represented once and enrichment values shown across all three IC levels. Red borders around heatmap cells indicate that the variant exceeded the enrichment threshold at that specific inhibitory concentration.*

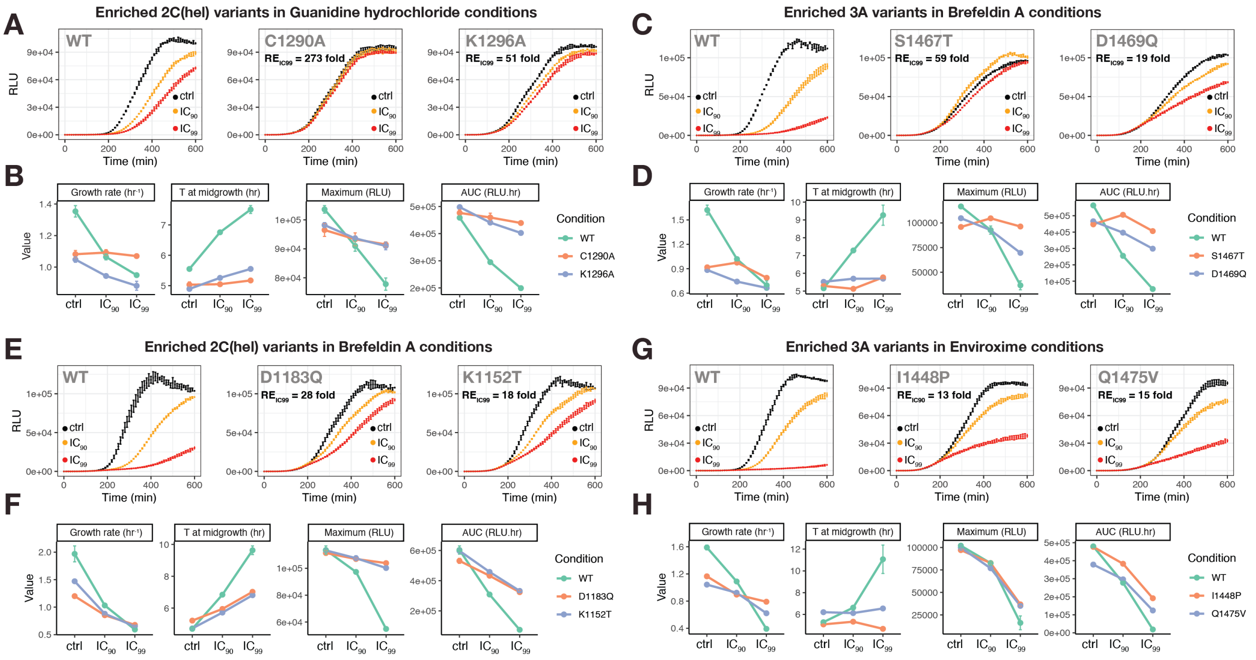

Supplementary Figure 5: Validation of Deep Mutational Scanning Data using Viral Replicon Assays. *EV-A71 replicon activity was measured as luminescence (relative light units, RLU) over time (minutes) and displayed as time-resolved growth measurements (points) with mean ± standard deviation (SD). For each condition, values represent the mean of three biological replicates.* ***(A)*** *Time-dependent replicon growth measurements for mutants enriched in the 2C condition in the presence of Guanidine hydrochloride.* ***(B)*** *Parameters derived from sigmoid fits to the growth data shown in (A) for wild-type and 2C mutants. The growth rate (r) corresponds to the maximal population growth rate at the steepest point of the curve. The time at mid-growth (t_mid) is defined as the time at which luminescence reaches half of the carrying capacity. The maximum signal (K) represents the carrying capacity, corresponding to the saturated maximal luminescence reached. AUC represents the empirical area under the curve of the luminescence signal over 10 hours. For each condition, parameters are reported as the mean ± SD from three biological replicates.* ***(C, D)*** *Same as (A, B) for mutants enriched in 3A under Brefeldin A treatment.* ***(E, F)*** *Same as (A, B) for 2C mutants enriched under Brefeldin A treatment.* ***(G, H)*** *Same as (A, B) for 3A variants enriched under Enviroxime treatment.*

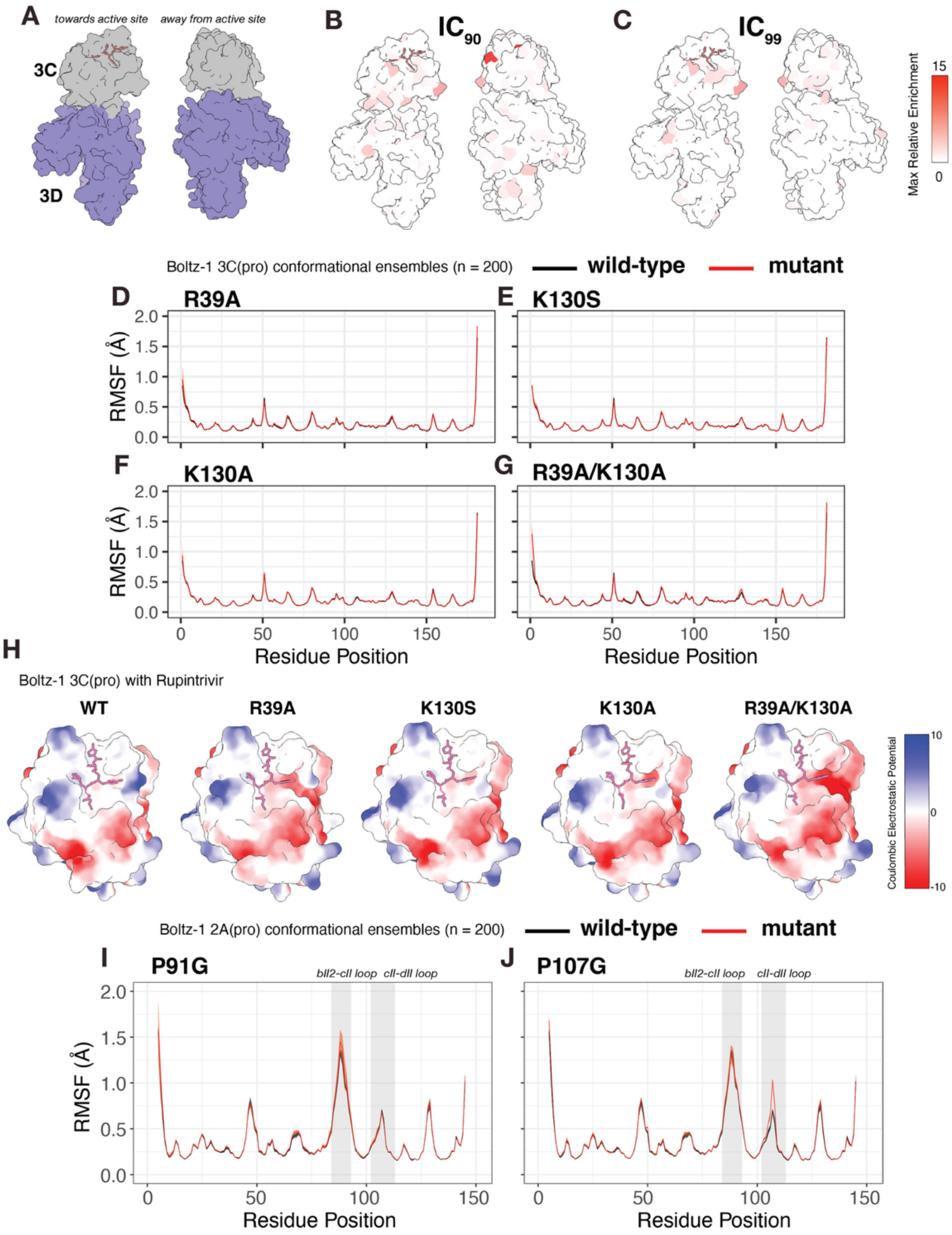

Supplementary Figure 6: Structural Impact of 2A and 3C protease Rupintrivir Enriched Mutations ***(A-C)*** *Structural model (Chai-1, pTM= 0.78, ipTM= 0.91) of the 3CD polyprotein precursor in complex with Rupintrivir (pink), showing the 3C domain in grey and the 3D domain in purple* ***(A)****. Rupintrivir-enriched mutations identified at* IC_{90} ***(B)*** *and* IC_{99} ***(C)*** *are mapped onto this complex. Line plots showing the Root Mean Square Fluctuation (RMSF) in Ångströms (Å) for wild-type 3C protease (black line) compared to mutant 3C proteases (red line):* ***(D)*** *R39A,* ***(E)*** *K130S,* ***(F)*** *K130A, and* ***(G)*** *Double mutant (DM) with R39 and K130A mutations. The standard deviation is calculated from three independent runs and shown using geom_ribbon in ggplot2.* ***(H)*** *Structural models (Boltz-1) of the wild type and mutant forms of the 3C protease with Rupintrivir are shown. Surfaces are colored according to Coulombic electrostatic potential, with red indicating negative charge and blue indicating positive charge. Rupintrivir is shown in pink. Line plots showing the RMSF in Å for wild-type 2A protease (black line) compared to mutant 2A proteases (red line):* ***(I)*** *P91G and* ***(J)*** *P107G. The standard deviation is calculated from three independent runs and shown using geom_ribbon in ggplot2. The bII2-cII and cII-dII loops are highlighted with gray boxes.*

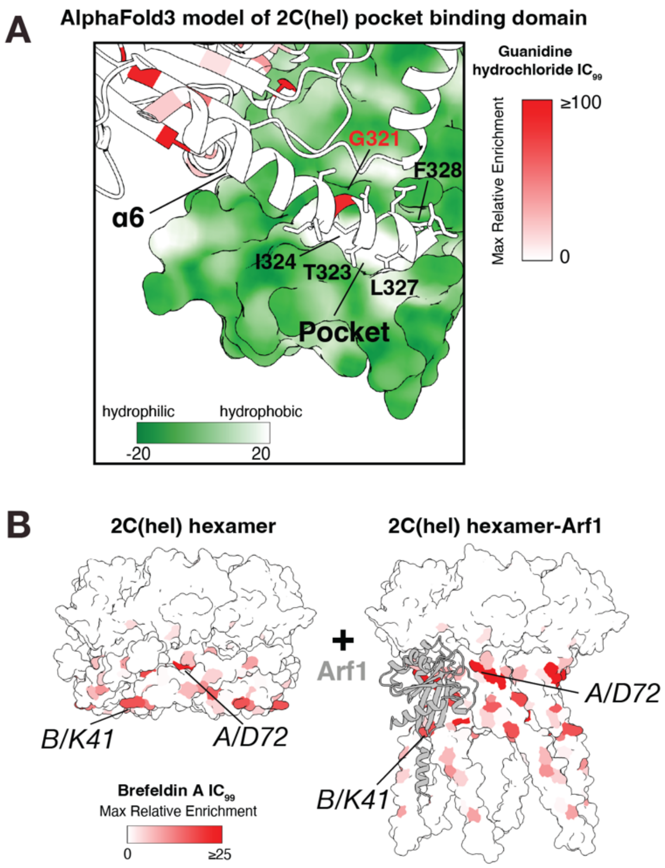

Supplementary Figure 7: **AlphaFold3 Models of the 2C hexamer and interaction with Arf1** ***(A)*** *Structural model showing the 2C multimer interactions. One 2C chain is colored according to the enrichment of mutations to Guanidine hydrochloride and the other chain (pocket) is colored by hydrophobicity.* ***(B)*** *Structural model showing the 2C multimer with or without Arf1. Annotated residues are in contact with Arf1 and overcome Brefeldin A inhibition.*

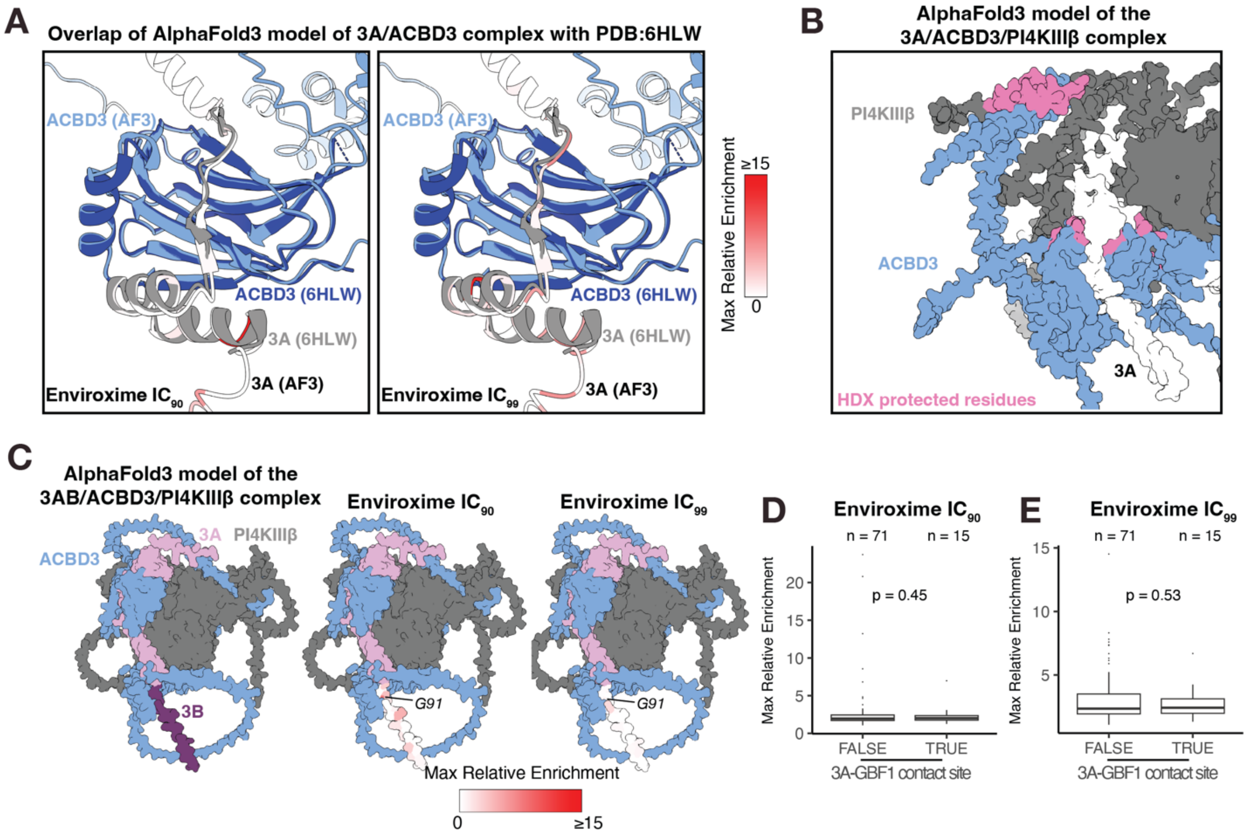

Supplementary Figure 8: Hydrogen/Deuterium Exchange Data Mapped onto the AlphaFold3 Model of the 3A/PI4KIIIβ/ACBD3 Complex and Enviroxime Enriched Mutants and GBF1 Contact Sites ***(A)*** *Structural similarity between the AlphaFold3 Model of the 3A/PI4KIIIβ/ACBD3 and the crystal structure of the EVA71 3A–ACBD3 GOLD domain complex (PDB:6HLW).* ***(B)*** *Residues protected from Hydrogen/Deuterium exchange are highlighted in pink. Data for highlighting residues was retrieved from McPhail et al.* (34) **(C)** *Model of the 3AB/PI4KIIIβ/ACBD3 complex mapping the Enviroxime enriched variants (AlphaFold3, pTM= 0.38, ipTM= 0.31).* ***(D-E)*** *Box plot comparing the max relative enrichment of Enviroxime mutations at 3A residue position that interact (TRUE) or do not interact (FALSE) with GBF1. Statistical significance was determined using a one-sided Wilcoxon-Mann-Whitney test.*
