# Supplementary material for: Genotype-by-inhibitor interactions to dissect enterovirus replication
Source: Nat Commun. 2026 Apr 11;17:5089. doi: 10.1038/s41467-026-71900-3 (PMC13247092; doi:10.1038/s41467-026-71900-3)
Supplement: Supplementary file 2 — Reporting summary [file 41467_2026_71900_MOESM2_ESM.pdf]

nature portfolio

Corresponding author(s): Patrick Dolan

Last updated by author(s): 2026-3-27

Reporting Summary

Nature Portfolio wishes to improve the reproducibility of the work that we publish. This form provides structure for consistency and transparency in reporting. For further information on Nature Portfolio policies, see our Editorial Policies and the Editorial Policy Checklist.

Statistics

For all statistical analyses, confirm that the following items are present in the figure legend, table legend, main text, or Methods section.

n/a	Confirmed	
☐	☒	The exact sample size (*n*) for each experimental group/condition, given as a discrete number and unit of measurement
☐	☒	A statement on whether measurements were taken from distinct samples or whether the same sample was measured repeatedly
☐	☒	The statistical test(s) used AND whether they are one- or two-sided<br>*Only common tests should be described solely by name; describe more complex techniques in the Methods section.*
☐	☒	A description of all covariates tested
☐	☒	A description of any assumptions or corrections, such as tests of normality and adjustment for multiple comparisons
☐	☒	A full description of the statistical parameters including central tendency (e.g. means) or other basic estimates (e.g. regression coefficient) AND variation (e.g. standard deviation) or associated estimates of uncertainty (e.g. confidence intervals)
☐	☒	For null hypothesis testing, the test statistic (e.g. *F*, *t*, *r*) with confidence intervals, effect sizes, degrees of freedom and *P* value noted<br>*Give P values as exact values whenever suitable.*
☐	☒	For Bayesian analysis, information on the choice of priors and Markov chain Monte Carlo settings
☐	☒	For hierarchical and complex designs, identification of the appropriate level for tests and full reporting of outcomes
☐	☒	Estimates of effect sizes (e.g. Cohen's *d*, Pearson's *r*), indicating how they were calculated

Our web collection on statistics for biologists contains articles on many of the points above.

Software and code

Policy information about availability of computer code

Data collection	*Provide a description of all commercial, open source and custom code used to collect the data in this study, specifying the version used OR state that no software was used.*
Data analysis	R version v4.3.3 (2024-02-29) "Angel Food Cake"<br>python v3.7<br>Boltz1 - v1<br>Chai1 - v0.6.1<br>ChimeraX - v1.11<br>AlphaFold3<br>R packages:<br>bio3D v2.4-5<br>tidyverse v2.0.0

For manuscripts utilizing custom algorithms or software that are central to the research but not yet described in published literature, software must be made available to editors and reviewers. We strongly encourage code deposition in a community repository (e.g. GitHub). See the Nature Portfolio guidelines for submitting code & software for further information.

Data

Policy information about availability of data

All manuscripts must include a data availability statement. This statement should provide the following information, where applicable:

- Accession codes, unique identifiers, or web links for publicly available datasets
- A description of any restrictions on data availability
- For clinical datasets or third party data, please ensure that the statement adheres to our policy

Code and analysis scripts used to generate the figures and analysis reported are available (https://github.com/QVEU/Genotype_Inhibitor_Interactions_Enterovirus_Replication). All raw sequencing read data is publicly available in the NCBI Short Read Archive, under project number PRJNA1404315.

Research involving human participants, their data, or biological material

Policy information about studies with human participants or human data. See also policy information about sex, gender (identity/presentation), and sexual orientation and race, ethnicity and racism.

Reporting on sex and gender	NA
Reporting on race, ethnicity, or other socially relevant groupings	NA
Population characteristics	NA
Recruitment	NA
Ethics oversight	NA

Note that full information on the approval of the study protocol must also be provided in the manuscript.

Field-specific reporting

Please select the one below that is the best fit for your research. If you are not sure, read the appropriate sections before making your selection.

☒ Life sciences ☐ Behavioural & social sciences ☐ Ecological, evolutionary & environmental sciences

For a reference copy of the document with all sections, see nature.com/documents/nr-reporting-summary-flat.pdf

Life sciences study design

All studies must disclose on these points even when the disclosure is negative.

Sample size	All sample sizes are reported where applicable. Sample sizes were chosen based on previous analysis of similar viral phenotypes, and prior work with the mutational library used here. Depending on assay, 3-4 replicates were included in validation experiments.
Data exclusions	No data excluded.
Replication	All experimental data is reported for replicates, with N reported. We found good correspondence between replicate experiments, as reported in manuscript.
Randomization	No randomization was performed.
Blinding	None applied. However, experimenters are only aware of outcome after sequencing analysis.

Reporting for specific materials, systems and methods

We require information from authors about some types of materials, experimental systems and methods used in many studies. Here, indicate whether each material, system or method listed is relevant to your study. If you are not sure if a list item applies to your research, read the appropriate section before selecting a response.

April 2023

Materials & experimental systems

n/a	Involved in the study
☒	☐ Antibodies
☐	☒ Eukaryotic cell lines
☒	☐ Palaeontology and archaeology
☒	☐ Animals and other organisms
☒	☐ Clinical data
☒	☐ Dual use research of concern
☒	☐ Plants

Methods

n/a	Involved in the study
☒	☐ ChIP-seq
☒	☐ Flow cytometry
☒	☐ MRI-based neuroimaging

Eukaryotic cell lines

Policy information about cell lines and Sex and Gender in Research

Cell line source(s)	ATCC (RD cells; CCL-136)
Authentication	Authenticated by genetic analysis.
Mycoplasma contamination	Confirmed negative by regular PCR testing
Commonly misidentified lines (See ICLAC register)	NA

Plants

Seed stocks	NA
Novel plant genotypes	NA
Authentication	NA
