# Supplementary material for: Genotype-by-inhibitor interactions to dissect enterovirus replication
Source: Nat Commun. 2026 Apr 11;17:5089. doi: 10.1038/s41467-026-71900-3 (PMC13247092; doi:10.1038/s41467-026-71900-3)
Supplement: Supplementary file 3 — Transparent Peer Review file [file 41467_2026_71900_MOESM3_ESM.pdf]

nature portfolio

Peer Review File

Genotype-by-Inhibitor Interactions to Dissect Enterovirus Replication

Corresponding Author: Dr Patrick Dolan

This file contains all reviewer reports in order by version, followed by all author rebuttals in order by version.

Version 0:

Reviewer comments:

Reviewer #1

(Remarks to the Author)
This is an outstanding manuscript, well written and considered, probing enterovirus biology using established pharmaceuticals, known and computer-generated structures, and deep sequencing to identify novel functional regions of viral proteins. The data are well presented and the interpretations clear and reasoned. The work is insightful, and the authors anchor their analyses in the biology of the virus, including demonstration of possible overlap functions of the two viral proteases, dividing the ATPase and replication organelle-protein recruitment functions of NSP-2C, and confirming the role of NSP-3A in recruiting PI4-Kinase to replication organelles through ACBD3 binding.

There is only one request for the authors; that is to reconsider their data in terms of the sequences and predicted structures of the immediate proteolytic precursors of their NSPs of interest, especially 3AB and 3CD. These intermediate precursors have reported functionality independent of their products. Looking at the data, it seems the Enviroxime data set is the most critical for a re-analysis, as the small 3B region seems to have some enrichment of mutations. The field has considered for years that 3AB may be the primary functional protein in many cases and these analyses may provide evidence for this in terms of the host factor interface. Rupintrivir mutational enrichment in 3C is relatively minor, so the changes in 3D which appear just below the threshold may be worth analyzing, particularly using co-variance analysis against the identifed 3C mutations to see if 3CD may be the functional protein.

Guanidine appears very specific to the intended target 2C, and the provided BFA analysis indicating overlapping protease function with 2A is sufficient, espcially since 3B appears to lack mutations. The latter two data sets therefore likely do not need re-analysis for 2BC and 3AB.

Overall this is a significant and excellent contribution to multiple fields of enterovirus inquiry.

Reviewer #2

(Remarks to the Author)
The authors present a large deep mutational scanning study of an enterovirus replication complex in the presence of 4 inhibitors of viral replication. This is an extension of their previous study published in Nat Microbiol. 2024 Nov 28;10(1):158–168, which determined the sequence requirements for viral replication fitness in RD cells. Noteworthy outcomes of the study presented in this manuscript are the discovery of compensatory escape variants that occur outside the inhibitor target protein in the viral genome and evidence for the strengthening of host factor interactions to escape trafficking defects introduced by BrefeldinA.
Runpintrivir targets 3C protease, but escape variants in the protease 2A were observed, in addition to those epected in 3C. The nature of the 2A mutations suggest a broadening of the 2A cleavage motif to include 3C substrate sequences. Biochemical studies to support this hypothesis were not performed. As 3C and 2A proteins can be produced recombinantly for in vitro assays, motif broadening could be assayed to strengthen this finding.
The authors confirm that BrefeldinA resistence is mediated by 2C binding to host factor Arf1 as proposed in https://doi.org/10.1371/journal.ppat.1011673 and present an Alphafold3 model that is supported by the DMS data. Structural studies to support this model were not done, but I think these should be outside the scope of this study.
The authors also compare escape profiles between BrefeldinA and enviroxime in the context of 3A mutations. 3A anchors to intracellular membranes and recruits host factors, including GBF1, ARF1, and ACBD3–PIK4IIIβ complexes, to remodel

Golgi- and ER-derived membranes into replication organelles. By binding GBF1 and perturbing ARF1-dependent trafficking, 3A both redirects and inhibits normal secretory pathway function. The ACBD3–PIK4IIIβ axis is targeted by enviroxime and the GBF1-ARF1 axis by BrefeldinA, thus the escape variants arising from each treatment should report of different functions. The authors predicted models of 3A-GBF1 and 3A-ACBD3/PIK4IIIβ to assess the escape mutations. They compare their 3A-ACBD3/PIK4IIIβ to HDX data published in ref 32, but do not assess it in the context of https://doi.org/10.1371/journal.ppat.1007962, which reports crystal structures of 3A-ACBD3.
Overall, the manuscript is at the cutting edge of deep mutational scanning approaches, measuring not just the consequences of sequence perturbations within a protein, but instead the consequences on the whole genome.
The data is interesting, however the paper is stripped to its bare bones and would benefit from more explanation of what is known regarding each protein's role and the mechanism of action of each drug.
There are no quality metrics associated with Alphafold models and in some cases (eg 3A-ACBD3) existing known structures are ignored leaving the reader to wonder if the Alphafold models were consistent with experimental data or contrasting.
The paper also does not describe how the library was created and sequenced in any detail, except to refer the reader to a previous publication [ref 10]. As passage 1 virus was used, this should differ from the initial library, in that it should be depleted for less fit variants. This aspect of the approach should be explained and a visualisation used to show coverage of the starting library. My understanding is that this library was not barcoded, and instead fragmented prior to Illumina sequencing? From this I assume that most reads were wild-type sequence and were discarded and synonymous wild-type used as normalisation factor? Some more detail regarding data processing would be helpful.

In Figure 1A, panel 1, there are many mutations in 2C observed in the low concentration inhibitor escape. Do the authors think they are noise because they are not observed in higher concentration selections?

Supp Fig 1, please provide replicate details in figure legend.

Reviewer #3

(Remarks to the Author)
Bakhache et al. manuscript combines deep mutational scanning of enterovirus replication complex proteins with pharmacological interventions to understand interactions between different replication complex domains. They perform experiments to select for mutations providing resistance to small molecular inhibitors and show that for several such inhibitors resistance mutations arise outside the direct protein target of that inhibitor. Using protein modeling they provide several hypotheses on the possible mechanism of these resistance mutations. The study is clearly written and provides novel results, although experimental validation for some of the hypotheses based on DMS and modeling results would make the paper stronger.

1. It would be great to have some sort of simple schematic in Fig. 1 that shows how the experiment is done so that one does not need to go to methods or previous paper to understand how experiment is set up.
2. Authors keep referring to normalization to control condition without explicitly saying what the control condition is (presumably no drug treatment?)
3. Fig.1 would be useful to have something that indicates if the same sites or positions get enriched at the different compound concentrations
4. Would it be possible to validate some of the enriched mutations as presumably providing drug resistance outside the DMS library?
5. Similarly to the point above, would it be possible to validate some of the modeling predictions in an experimental setting? E.g. can you show that K41 or D72 2C variants are good at recruiting Arf1 in the absence of GBF1 using some microscopy based approach?

Reviewer #4

(Remarks to the Author)
I co-reviewed this manuscript with one of the reviewers who provided the listed reports. This is part of the Nature Communications initiative to facilitate training in peer review and to provide appropriate recognition for Early Career Researchers who co-review manuscripts.

Version 1:

Reviewer comments:

Reviewer #1

(Remarks to the Author)
The additional analyses are extremely interesting and the new 3B interface data add much to the manuscript. It is understandable that covariance analysis is not possible with the current dataset. There are no additional comments regarding this excellent manuscript.

Reviewer #2

(Remarks to the Author)
The authors have addressed my comments and updated the manuscript appropriately.

Reviewer #3

(Remarks to the Author)
I'm satisfied with authors' responses to my comments.

Reviewer #4

(Remarks to the Author)
I co-reviewed this manuscript with one of the reviewers who provided the listed reports. This is part of the Nature Communications initiative to facilitate training in peer review and to provide appropriate recognition for Early Career Researchers who co-review manuscripts.

Open Access This Peer Review File is licensed under a Creative Commons Attribution 4.0 International License, which permits use, sharing, adaptation, distribution and reproduction in any medium or format, as long as you give appropriate credit to the original author(s) and the source, provide a link to the Creative Commons license, and indicate if changes were made.
In cases where reviewers are anonymous, credit should be given to 'Anonymous Referee' and the source.
The images or other third party material in this Peer Review File are included in the article’s Creative Commons license, unless indicated otherwise in a credit line to the material. If material is not included in the article’s Creative Commons license and your intended use is not permitted by statutory regulation or exceeds the permitted use, you will need to obtain permission directly from the copyright holder.
To view a copy of this license, visit https://creativecommons.org/licenses/by/4.0/

Reviewer #1 (Remarks to the Author) This is an outstanding manuscript, well written and considered, probing enterovirus biology using established pharmaceuticals, known and computer-generated structures, and deep sequencing to identify novel functional regions of viral proteins. The data are well presented and the interpretations clear and reasoned. The work is insightful, and the authors anchor their analyses in the biology of the virus, including demonstration of possible overlap functions of the two viral proteases, dividing the ATPase and replication organelle-protein recruitment functions of NSP-2C, and confirming the role of NSP-3A in recruiting PI4-Kinase to replication organelles through ACBD3 binding.

There is only one request for the authors; that is to reconsider their data in terms of the sequences and predicted structures of the immediate proteolytic precursors of their NSPs of interest, especially 3AB and 3CD. These intermediate precursors have reported functionality independent of their products. Looking at the data, it seems the Enviroxime data set is the most critical for a re-analysis, as the small 3B region seems to have some enrichment of mutations. The field has considered for years that 3AB may be the primary functional protein in many cases and these analyses may provide evidence for this in terms of the host factor interface. Rupintrivir mutational enrichment in 3C is relatively minor, so the changes in 3D which appear just below the threshold may be worth analyzing, particularly using co-variance analysis against the identified 3C mutations to see if 3CD may be the functional protein. Guanidine appears very specific to the intended target 2C, and the provided BFA analysis indicating overlapping protease function with 2A is sufficient, especially since 3B appears to lack mutations. The latter two data sets therefore likely do not need re-analysis for 2BC and 3AB. Overall this is a significant and excellent contribution to multiple fields of enterovirus inquiry.

We thank the reviewer for this insightful suggestion. We agree that analyzing our data in the context of proteolytic precursors is a valuable and underexplored aspect of enterovirus replication. Following this advice, we performed new analyses on both the 3AB and 3CD precursors.

<u>Analysis of the 3AB Precursor and the Enviroxime Dataset</u> *As the reviewer correctly indicated, 3AB and 3A have distinct functions during viral replication (Jackson and Belsham 2021). To investigate this in the context of host factor interactions, we generated a new structural model (AlphaFold3) of 3AB in a complex with PI4KIIIβ and ACBD3 (Supp. Fig. 8C).*

This model revealed a novel interface where the 3B portion of 3AB directly contacts ACBD3. When we mapped our Enviroxime-enriched mutations onto this complex, we observed an enrichment of mutations at this newly identified 3B contact site, specifically glycine at position 91 in the IC90 condition (Supp. Fig. 8C). This finding supports the reviewer's suggestion that precursor forms like 3AB contribute to functional interactions at the host factor interface.

<u>Analysis of the 3CD Precursor and the Rupintrivir Dataset</u> *We also generated a structural model of 3CD in complex with Rupintrivir (Chai-1) and mapped the corresponding mutations (Supp. Fig. 6A-C). In this case, we did not observe a clear structural clustering of mutations in the 3D region or near the 3CD interface.*

Regarding the reviewer's suggestion to perform a co-variance analysis, this is not possible with our current dataset. Our method is based on short-read sequencing, which does not preserve the genetic linkage between distant sites like 3C and 3D. We have added a note to the discussion

(Lines 349-354) to highlight this limitation and to suggest that future studies using long-read sequencing could capture such linkage across the polyprotein.

We thank the reviewer again for this constructive feedback, as it has led to a significant new insight for our work.

Reviewer #2 (Remarks to the Author): The authors present a large deep mutational scanning study of an enterovirus replication complex in the presence of 4 inhibitors of viral replication. This is an extension of their previous study published in Nat Microbiol. 2024 Nov 28;10(1):158–168, which determined the sequence requirements for viral replication fitness in RD cells. Noteworthy outcomes of the study presented in this manuscript are the discovery of compensatory escape variants that occur outside the inhibitor target protein in the viral genome and evidence for the strengthening of host factor interactions to escape trafficking defects introduced by BrefeldinA.
Runpintrivir targets 3C protease, but escape variants in the protease 2A were observed, in addition to those expected in 3C. The nature of the 2A mutations suggest a broadening of the 2A cleavage motif to include 3C substrate sequences. Biochemical studies to support this hypothesis were not performed. As 3C and 2A proteins can be produced recombinantly for in vitro assays, motif broadening could be assayed to strengthen this finding.

We thank the reviewer for this suggestion. We agree that a detailed in vitro biochemical characterization of recombinant 2A and 3C proteases would be a powerful approach to directly test the hypothesis that certain 2A mutations broaden substrate specificity to include 3C-like cleavage motifs. While such biochemical studies represent an important future direction, we believe they are beyond the scope of the current manuscript which focuses on combining high-throughput genetics, perturbations, and structural modeling for dissecting replication mechanisms. We mention in the Results the value of future biochemical assays to explore this possible mechanism in greater detail (Lines 195–196).

However, in direct response to this comment, we have now performed functional validation experiments to test whether mutations enriched in our DMS screens confer inhibitor-specific phenotypes in an independent assay system. Specifically, we introduced a subset of enriched mutations (n = 8) into an EV-A71 NanoLuc replicon (Bakhache et al. 2025) and assessed replication kinetics in the presence of the corresponding inhibitors (Supplementary Figure 5).

Across conditions tested, mutations enriched in the DMS screen consistently supported replication under inhibitory conditions, and their phenotypic effects closely correlated with their relative enrichment values derived from the screen.

We further characterized replication kinetics in the tested conditions using growthcurver, an R package that fits a logistic function to the data to derive parameters such as growth rate, area under the curve, and time to the inflection point. Mutations enriched under guanidine hydrochloride selection altered early replication kinetics, entering the exponential phase more rapidly than wild type, consistent with enhanced efficiency of negative-strand RNA synthesis. In contrast, mutations enriched under Brefeldin A selection did not accelerate early replication kinetics but instead supported sustained replication in the presence of the inhibitor, consistent

with a role for GBF1 in replication organelle formation rather than negative-strand RNA synthesis. Notably, we further confirmed that mutations in 2C alone were sufficient to rescue replication at the IC99 concentration of Brefeldin A, providing additional evidence that alterations in this viral protein can overcome inhibition of the 3A–host factor interaction.

Together, these new functional data demonstrate that DMS enrichment reliably captures inhibitor-specific effects on viral replication in an independent experimental system. While not a substitute for future biochemical assays, these results provide strong functional support for our conclusions and establish a path for interpreting how mutations and inhibitors modulate distinct replication parameters.

The authors confirm that BrefeldinA resistence is mediated by 2C binding to host factor Arf1 as proposed in https://doi.org/10.1371/journal.ppat.1011673 and present an Alphafold3 model that is supported by the DMS data. Structural studies to support this model were not done, but I think these should be outside the scope of this study.
The authors also compare escape profiles between BrefeldinA and enviroxime in the context of 3A mutations. 3A anchors to intracellular membranes and recruits host factors, including GBF1, ARF1, and ACBD3–PIK4IIIβ complexes, to remodel Golgi- and ER-derived membranes into replication organelles. By binding GBF1 and perturbing ARF1-dependent trafficking, 3A both redirects and inhibits normal secretory pathway function. The ACBD3–PIK4IIIβ axis is targeted by enviroxime and the GBF1-ARF1 axis by BrefeldinA, thus the escape variants arising from each treatment should report of different functions. The authors predicted models of 3A-GBF1 and 3A-ACBD3/PIK4IIIβ to assess the escape mutations. They compare their 3A-ACBD3/PIK4IIIβ to HDX data published in ref 32, but do not assess it in the context of https://doi.org/10.1371/journal.ppat.1007962, which reports crystal structures of 3A-ACBD3.

We thank the reviewer for highlighting this crystal structure of the EVA71 3A–ACBD3 GOLD domain complex (PDB: 6HLW). We apologize for this oversight. We have now included a direct comparison between this crystal structure and our predicted model in Supplementary Figure 8A.

The comparison reveals high structural similarity between the experimentally determined structure and our computationally predicted model. This new analysis further strengthens the validity of our model and the conclusions we draw from it regarding 3A's interactions.

Overall, the manuscript is at the cutting edge of deep mutational scanning approaches, measuring not just the consequences of sequence perturbations within a protein, but instead the consequences on the whole genome.
The data is interesting, however the paper is stripped to its bare bones and would benefit from more explanation of what is known regarding each protein's role and the mechanism of action of each drug.

Thank you for this constructive feedback. We agree that the manuscript would benefit from a more detailed background to better frame our findings. In response, we have expanded the Introduction and the beginning of the Results sections. These revised sections now provide a more

comprehensive overview of the known functions of the viral proteins and the established mechanisms of action for each of the four inhibitors studied. We believe these additions now provide the necessary context for our DMS data and subsequent analyses.

There are no quality metrics associated with Alphafold models and in some cases (eg 3A-ACBD3) existing known structures are ignored leaving the reader to wonder if the Alphafold models were consistent with experimental data or contrasting.

We apologize for the omission of this information in the original submission. We have now updated all figure legends associated with structural prediction models to include standard confidence metrics, specifically the predicted Template Modeling score (pTM) and the inter-chain predicted Template Modeling score (iPTM). In addition, our GitHub repository includes all predicted models together with their corresponding JSON metric files.

The paper also does not describe how the library was created and sequenced in any detail, except to refer the reader to a previous publication [ref 10]. As passage 1 virus was used, this should differ from the initial library, in that it should be depleted for less fit variants. This aspect of the approach should be explained and a visualisation used to show coverage of the starting library. My understanding is that this library was not barcoded, and instead fragmented prior to Illumina sequencing? From this I assume that most reads were wild-type sequence and were discarded and synonymous wild-type used as normalisation factor? Some more detail regarding data processing would be helpful.

We sincerely apologize for the lack of clarity and for relying on our previous publication. We have improved the methods section and Figure 1 to better visualize the experimental pipeline used.

To address the reviewer's specific questions:

Library Passaging: The reviewer is correct. We used the "passage 1" (P1) virus population as the starting material for all drug selections. This is a critical step because the initial P0 library (generated from plasmid transfection) can be heavily affected by complementation. By performing one passage at a low MOI (to generate P1), we deplete the library of non-viable variants that would otherwise remain via complementation. This provides a more stable and functionally relevant starting population. In this study, this P1 population was then passaged again (to P2) in parallel, in the presence or absence of each inhibitor. We have clarified this experimental workflow in the text and added Supplementary Figure 1 to visualize the variant distribution of the plasmid input library.

Sequencing & Data Processing: The reviewer's understanding is correct. The library was not barcoded; we used a fragmentation-based sequencing approach. Regarding normalization, we did not apply a synonymous mutation normalization for this specific analysis. Our primary goal was to determine how mutational effects change in the presence of inhibitors. Therefore, we calculated relative enrichment scores by comparing drug-treated populations directly against the

no-drug control population. We have now explicitly detailed our data processing workflow in the Methods and in Figure 1B.

In Figure 1A, panel 1, there are many mutations in 2C observed in the low concentration inhibitor escape. Do the authors think they are noise because they are not observed in higher concentration selections?

We thank the reviewer for raising this point. Many of the mutations enriched at low inhibitory concentrations are also enriched at higher concentrations in panel A under Guanidine hydrochloride treatment. Therefore, these variants are not simply background noise specific to low-stringency selection. This shared enrichment pattern is more evident in our DMS-by-condition heatmaps (Supplementary Figure 4), which directly compare mutation effects across inhibitory concentrations. To avoid confusion, we have now added a clarifying sentence to the main text in the section discussing Supplementary Figure 4 to explicitly highlight this consistency across conditions.

Supp Fig 1, please provide replicate details in figure legend.

We have now updated the legend to include details on the replicates for this figure (now Supplementary Figure 3). Additionally, we added new panels showing the inter-replicate correlation analysis for the passaging experiments, which shows the high reproducibility of the DMS data.

Reviewer #3 (Remarks to the Author): Bakhache et al. manuscript combines deep mutational scanning of enterovirus replication complex proteins with pharmacological interventions to understand interactions between different replication complex domains. They perform experiments to select for mutations providing resistance to small molecular inhibitors and show that for several such inhibitors resistance mutations arise outside the direct protein target of that inhibitor. Using protein modeling they provide several hypotheses on the possible mechanism of these resistance mutations. The study is clearly written and provides novel results, although experimental validation for some of the hypotheses based on DMS and modeling results would make the paper stronger.

1. It would be great to have some sort of simple schematic in Fig. 1 that shows how the experiment is done so that one does not need to go to methods or previous paper to understand how experiment is set up.

We thank the reviewer for this excellent suggestion. We agree that a visual schematic of the experimental setup would significantly improve clarity. We have now revised Figure 1 to include a new schematic diagram that illustrates the complete experimental and data analysis workflow, including the passaging of the DMS library in the presence of the inhibitors. We believe this addition makes the figure more self-contained and easier to interpret.

2. Authors keep referring to normalization to control condition without explicitly saying what the control condition is (presumably no drug treatment?)

We thank the reviewer for pointing out this ambiguity. The reviewer is correct in their assumption. We have now clarified in the text that the "control condition" is the parallel no-drug treatment population. Furthermore, our revised Figure 1 schematic (added in response to Point 1) now visually illustrates this experimental design.

3. Fig.1 would be useful to have something that indicates if the same sites or positions get enriched at the different compound concentrations

We thank the reviewer for this helpful suggestion. We agree that this is an important point. We refer to this pattern in our DMS-by-condition heatmaps in Supplementary Figure 4*, which allow for a direct comparison of enriched sites across the different compound concentrations. To make this clearer for the reader, we have now added a sentence to the main text (when discussing* Supplementary Figure 4*) to explicitly clarify this point and direct the reader to the relevant heatmaps.*

4. Would it be possible to validate some of the enriched mutations as presumably providing drug resistance outside the DMS library?
5. Similarly to the point above, would it be possible to validate some of the modeling predictions in an experimental setting? E.g. can you show that K41 or D72 2C variants are good at recruiting Arf1 in the absence of GBF1 using some microscopy based approach?

We thank the reviewer for these insightful suggestions and agree that independent validation of enriched mutations and modeling-based hypotheses is critical for interpreting the DMS data.

In direct response to point (4), we have now performed functional validation experiments to test whether mutations enriched in our DMS screens confer inhibitor-specific phenotypes outside the pooled library context. Specifically, we introduced a subset of enriched mutations (n = 8) into an EV-A71 NanoLuc replicon system and assessed replication in the presence of the corresponding inhibitors (Supplementary Figure 5). Across conditions tested, mutations enriched in the DMS screens consistently supported replication under inhibitory conditions, and their phenotypic effects correlated closely with their enrichment scores. These results demonstrate that DMS enrichment reliably identifies mutations that allow viral replication under inhibitory conditions in an independent assay system.

In response to point (5), we additionally performed targeted validation of 2C mutations implicated by our structural models. Specifically, we introduced the K41 and D72 substitutions into the replicon background and tested their ability to support replication under Brefeldin A treatment. Under IC99 inhibitor conditions, these 2C mutations were sufficient to restore robust replication despite inhibition of the 3A–host factor pathway (Supplementary Figure 5E and F). These results indicate that specific 2C substitutions can functionally compensate for disruption of GBF1-dependent replication, consistent with a bypass or rewiring of the Arf1-associated pathway. Notably, these findings build on prior work by Dr. George Belov's lab (Viktorova et al. 2023) ,

which identified related regions of 2C as important for overcoming Brefeldin A inhibition in poliovirus.

While direct imaging-based visualization of Arf1 recruitment by individual 2C mutants would be valuable, such experiments are beyond the scope of the current revision, which focuses on demonstrating how combining DMS with structural modeling can lead to mechanistic insight into viral replication.

References

Bakhache, William, Ann Shen, Walker Symonds-Orr, Megan Culler Freeman, and Patrick T. Dolan. 2025. "Novel Reporter Constructs to Accelerate Antiviral and Therapeutic Discovery for Enterovirus-A71." *Antiviral Research* 235 (106094): 106094.

Jackson, Terry, and Graham J. Belsham. 2021. "Picornaviruses: A View from 3A." *Viruses* 13 (3): 456.

Viktorova, Ekaterina G., Samuel Gabaglio, Seyedehmahsa Moghimi, et al. 2023. "The Development of Resistance to an Inhibitor of a Cellular Protein Reveals a Critical Interaction between the Enterovirus Protein 2C and a Small GTPase Arf1." *PLoS Pathogens* 19 (9): e1011673.
